# Supplementary material for: Comparison of COVID-19 testing strategies and costs for professional sports teams: A case study of J. League clubs
Source: PLoS One. 2025 Apr 7;20(4):e0310939. doi: 10.1371/journal.pone.0310939 (PMC11975107; doi:10.1371/journal.pone.0310939)
Supplement: S1 Appendix — (DOCX) [file pone.0310939.s002.docx]

**S2 Appendix 1: Total cost when revenue loss is 10 times the value assumed in the main text.**

We determined the total infection cost with a revenue loss of 14,000,000 yen/person (10 times the value used in the text, to reflect the above-average incomes of professional players or staff).

Fig S2A and S2B show the total costs under the regular-testing scenarios. When *P*_0_ is small (10^–5^), the total costs in Fig S2A are slightly higher than those in Fig 6A in the main text. When *P*_0_ is large (10^–3^), the total costs in Fig S2B are roughly five times those in Fig 6B in the main text.

Fig S2C and S2D show the total costs under the additional-testing scenarios. When *R*_0_ = 2.5, the order of costs is roughly the same as that obtained in the main text (Fig 6C); but in Fig. S2C, the cost of scenario O-P-1d-0d (daily PCR testing with a reading time of 0 days) is the second lowest, whereas in Fig 6C in the main text, the cost of scenario O-P-1d-0d is the fourth lowest. When *R*_0_ = 5.0, scenario O-A-1d-0d (daily antigen testing) has the lowest cost in Fig 6D in the main text, but scenario O-P-1d-0d (daily PCR testing with a reading time of 0 days) has the lowest cost in Fig S2D. These results indicate that, when the cost of lost opportunities is high, the total cost is less in the case of the more sensitive PCR test.


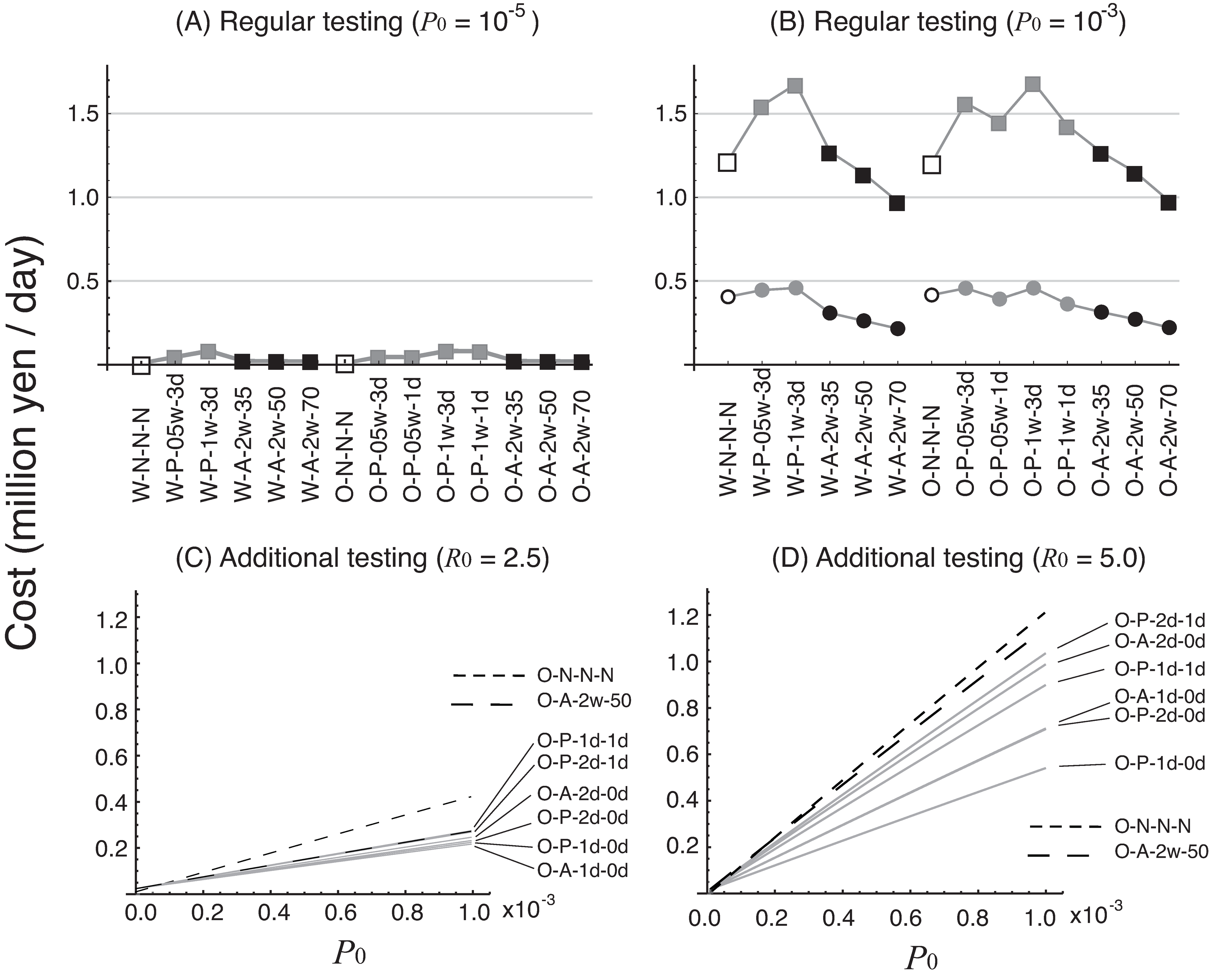


**Fig S2. Cost of testing scenarios.**

Cost of testing scenarios when the revenue loss due to the quarantine of infected players or staff (*Iv*) = 1.4 million yen/person: (A) regular-testing scenarios (*P*_0_ = 10^–5^), (B) regular-testing scenarios (*P*_0_ = 10^–3^), (C) additional-testing scenarios (*R*_0_ = 2.5), (D) additional-testing scenarios (*R*_0_ = 2.5). *P*_0_ is the individual infection rate per day (level of community transmission). In (A) and (B), the lower and upper lines represent *R*_0_ = 2.5 and 5, respectively. The two lines overlap in A because of the similar values. With the exception of O-N-N-N, the order of scenarios regarding costs remains the same, irrespective of *P*_0_.
