# Supplementary material for: Comparison of COVID-19 testing strategies and costs for professional sports teams: A case study of J. League clubs
Source: PLoS One. 2025 Apr 7;20(4):e0310939. doi: 10.1371/journal.pone.0310939 (PMC11975107; doi:10.1371/journal.pone.0310939)
Supplement: S2 Appendix — (DOCX) [file pone.0310939.s003.docx]

**S3 Appendix 2 The process of estimating the basic reproduction number from the number of infected individuals in a population of 50 individuals.**

Deviation in FES was observed between the continuous population-size model (Eq. 1 in the main text) and the discrete population-size model (Fig S4 in S4 Appendix 3), for the simple reason that the population size was based on the discrete population-size model. As noted in the main text (in the section, “Cautionary note regarding average-based evaluation”), when *R*_0_ = 5.0, the FES distribution has two peaks (right panel in Fig. 8), which are never observed in the continuous population-size model.

Fig S3 shows the FES with no infection-control measures (no testing or daily symptom check). In the left panel (*R*_0_ = 2.5) there are roughly 2000 cases where only one infected individual (the first one to enter the group) was detected before the cessation of infection; that is, 2000 cases involved no further infection.

Whether or not new infection occurs is a probability event in the discrete population model, and chance plays a role; as *R*_0_ increases, however, the stronger force of infection outweighs the chance effects, and the number of cases in which secondary infection does not occur by chance decreases. Thus, when *R*_0_ = 5.0 (right panel in Fig S3), there are roughly 1000 cases involving no secondary infection—fewer than for *R*_0_ = 2.5. As the number of infected individuals increases, it becomes less likely that all infected individuals will recover at the same time, and the number of infected individuals thereby approaches the value of FES determined by *R*_0_ (the distribution peak where FES is high).


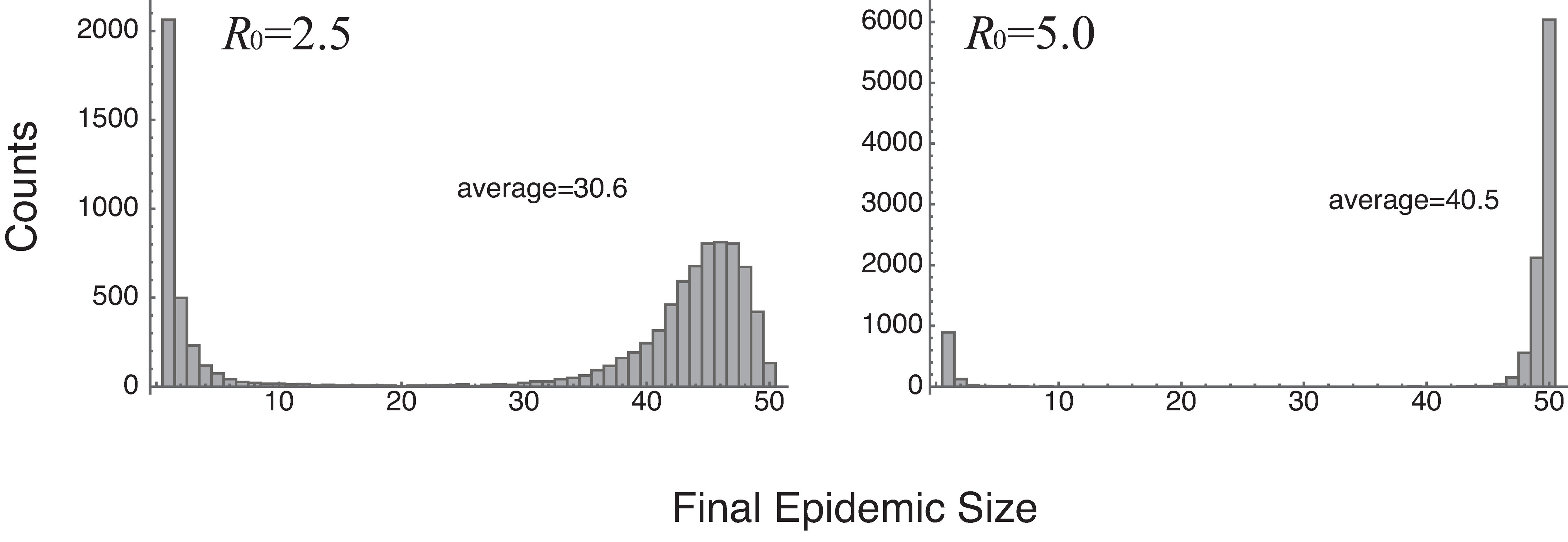


**Fig S3. Histogram of the final epidemic size with no infection-control measures, in 10,000 Monte-Carlo iterations .**

In reality, such chance will typically be at work in small populations. When we calculate the mean of infected individuals, we do so on the basis of the observed events, and (as aforementioned) there will be cases in which further infections occur infrequently and no infected individuals are actually detected in the population. As the average values derived from observation exclude these cases, such values may be underestimated.

Whether the final number of infections will be higher or lower than average is determined by chance in small populations. When we encounter mass infection in a group, we are often concerned about the possibility of inadequate infection control; however, although this possibility cannot be ignored, there is also the possibility that we are simply unlucky in the given case.
