# Supplementary material for: Comparison of COVID-19 testing strategies and costs for professional sports teams: A case study of J. League clubs
Source: PLoS One. 2025 Apr 7;20(4):e0310939. doi: 10.1371/journal.pone.0310939 (PMC11975107; doi:10.1371/journal.pone.0310939)
Supplement: S3 Appendix — (DOCX) [file pone.0310939.s004.docx]

**S4 Appendix 3. Reproductive number under the testing scenarios**

The cumulative number of infected individuals occurring in a given population until the cessation of infection is termed the final epidemic size (FES) and is determined by the basic growth rate (*R*_0_). This means that, if the FES in a given population is known, the *R*_0_ for that infection can be determined.

First, then, we obtain FES as a function of *R*_0_ when no control measures are taken (no testing or daily symptom checks). Fig S4 shows the FES obtained (dashed line) by solving a continuous-time and continuous population-size model (Eq. 1 in the main text) and (solid line) by solving a discrete population size model by using Monte-Carlo simulations (all other results in the main text). These two results would be similar in a large population, but they diverge for the groups of 50 individuals investigated in this study. (See also S3 Appendix 2.)

We then solve the infection dynamics under a given testing scenario to obtain the relevant FES. Once the FES is obtained, we select the FES closest to the solid line in Fig S4 and find the corresponding value of *R*_0_, which is the reproductive number that includes the effects of the given testing scenario and is termed the effective reproductive number (*Re*).


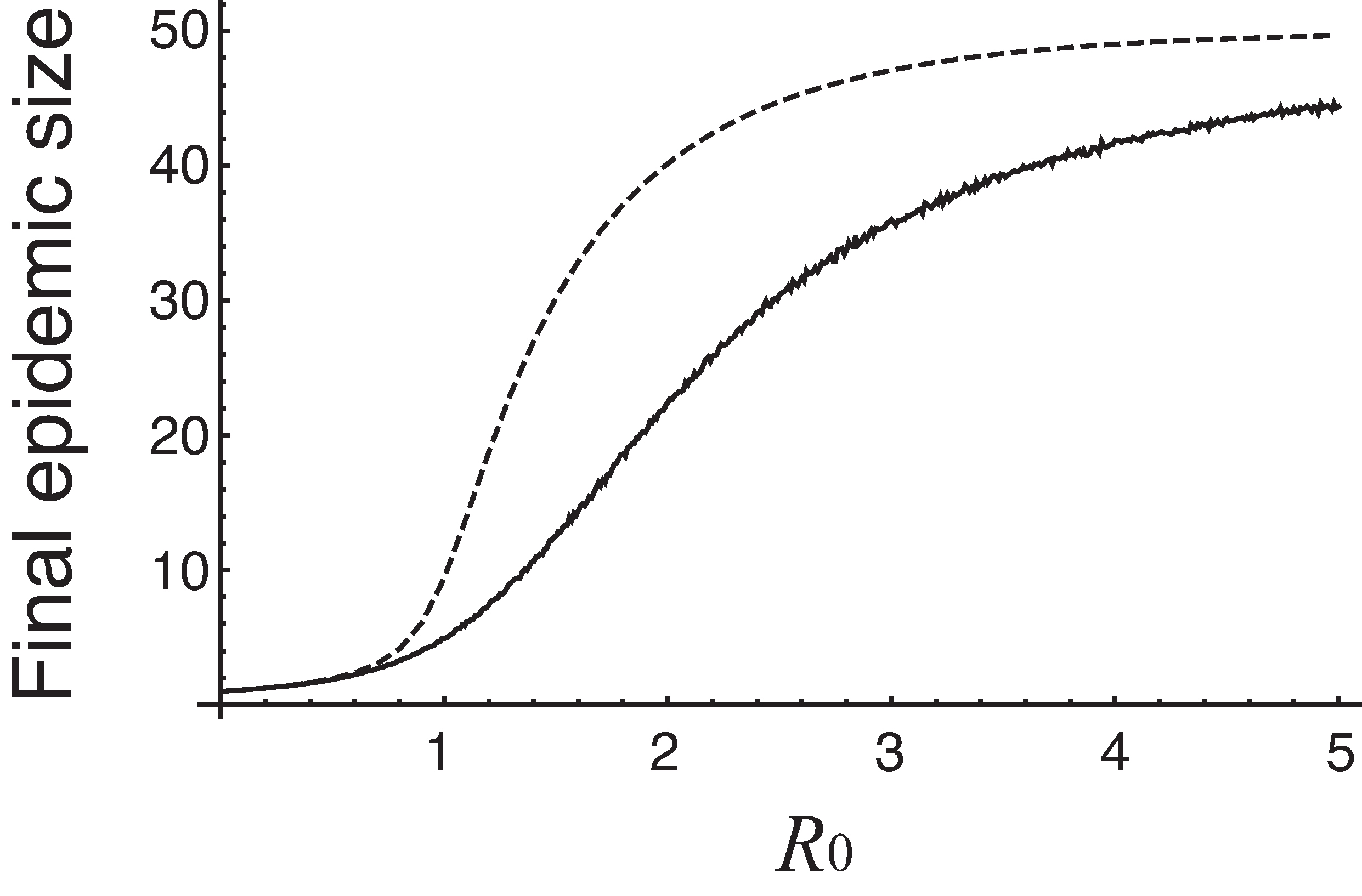


**Fig S4. Final epidemic size (FES) versus basic growth rate (*R*_0_) without quarantining of infected individuals.**

The dashed line shows the FES obtained by Eq. 1 in the main text. The solid line shows the FES obtained by the Monte-Carlo simulations, as the mean of 10,000 iterations. In the model, the time is continuous but the population size is discrete; as the Monte-Carlo simulation is a stochastic process, the line does not become completely smooth.
