# Supplementary material for: Comparison of COVID-19 testing strategies and costs for professional sports teams: A case study of J. League clubs
Source: PLoS One. 2025 Apr 7;20(4):e0310939. doi: 10.1371/journal.pone.0310939 (PMC11975107; doi:10.1371/journal.pone.0310939)
Supplement: Attachment — Submitted filename: Response_to_reviewers.docx [file pone.0310939.s006.docx]

Reviewer #1: Dear Dr. Kamo and research group members

This paper attempts to establish a mathematical model to describe the impact of COVID-19 testing methods on the spread and control of internal infections within a finite group. It also discusses the relevant cost-effectiveness relationship. I appreciate the contributions of all authors and believe This paper should be published due to its instructive value to occupational health and public health.

However, I have some concerns and believe that the paper requires further revision.

[Our reply]

We would like to thank the reviewer for taking the time to review our paper.

Major Revision Suggestions

1. Definition of Recovered (R): On page 4, the authors define “recovered (R)”, but it is not discussed in the subsequent model. It is important to note that newly infected and reinfected individuals often have different susceptibilities to the virus. If in this paper S=R, this should be clearly stated.

[our reply]

In this paper, we assume a small population and simulate a short period of time in which an infectious disease spreads from a single infected individual to the population until it is eradicated within the population. With such a short time interval, we can ignore the mutation of the virus, so we assume that recovered individuals will never be infected again. We also ignore the birth and death process, so the total number of individuals in the population will be constant. The number of individuals in state R will be determined once the number of individuals in states other than R is determined, so we did not particularly discuss the number of individuals in the text. We now state the assumption that we are considering a very short time interval and that the immunity acquired by R is complete and that R will never be infected again (page 4, lines 115-117).

2. PCR Reading Time: On page 7, the authors state that the PCR reading time is 1 to 3 days. Meanwhile, on page 9, they claim that a wait of 0 days is unrealistic. However, in the mid-to-late and post-epidemic era, PCR results typically take only a few hours to half a day. Given that this paper will be published in a post-epidemic context, this expression may need adjustment.

[Our reply]

We initially referred to the time it takes to get results as the reading time, but this was not very appropriate. The reading time refers to the time taken to collect the sample, send it to the testing laboratory, and obtain the results. In the additional-testing scenario, which is a kind of kind of emergency for sports teams, we assumed that a rapid testing regime would be used. We have now made it clear that the reading time indicated includes not only the actual reading time for PCR tests but also the turnaround time for obtaining the test results (page 8, lines 213-216, page 10 lines 260-262).

3. Figure Confusion: In Figure 3A, it is unclear why the antigen testing scenarios is included in the PCR testing figure. The representations of the black circle and square do not seem as 0 value. The similar issue also appears in other figures.

[Our reply]

The reason for the non-zero number for PCR testing in the antigen-testing scenarios is that, in the J.League clubs, PCR testing was performed on individuals that had tested positive by antigen testing, as we explained in our earlier version. As the reviewer points out, this issue can confuse readers, so we have added the reason in the caption to Figure 3 (now Figure 4). Please also see on page 11, lines 300-304.

4. Due to this paper will be published in the post-epidemic era the infection protection policies have changed fundamentally, the authors should discussion the making to the unknown epidemic in the future and policy implications form this research.

[Our reply]

This is a very important comment. Efficiency of the testing system (including cost-effectiveness) is essential for the management of infectious diseases. The efficiency is determined on the basis of various factors (basic reproduction number, sensitivity of the test, cost of the test). It is rare that all of these factors are known immediately after the emergence of a new infectious disease. A simulator that can flexibly incorporate factors that affect the testing efficiency is extremely important. We believe that our study is important in providing a basis to the arguments for different test regimes. However (and in relation to the next comment), our study had limitations. We examined the efficiency of testing regimes in small groups. In addition, we examined the period from the start of infection to the end, but this was possible because we considered only a small group. In the case of large groups, it is very difficult to eradicate infectious diseases, and in many cases the infectious disease dynamics reach an endemic state. If the infectious disease continues for a long time, we cannot ignore the reinfection of recovered individuals by mutants, as well as the birth and death process in the population dynamics. Our model is difficult to adapt to such long-term infectious disease countermeasures. We have now revised the text to include these arguments (page 25, lines 661-671, page 29, lines 800-821).

5. Limitations: The authors should clearly state the limitations of this research.

[Our reply]

Please see [Our reply] to comment #4.

Minor Revise suggestions

1. Author and Institution ID: The ID of authors and institution affiliations do not match; please verify for consistency.

[Our reply]

Thank you for pointing this out. We will check it carefully when we submit the paper.

Reviewer #2: Abstract

The abstract is clear and concise, providing a good overview of the study’s focus on infection control in professional sports teams. The use of the SEIR model is well-articulated, and the distinction between the two testing scenarios (regular and additional testing) is clear. The abstract effectively highlights the main outcomes, showing that antigen testing is more efficient than PCR testing in terms of costs and infection reduction.

[Our reply]

First of all, we would like to thank you for the time you have taken to read our paper. We also thank you for your many suggestions. Please see our line-by-line replies below.

Suggestions:

• The abstract could start with a brief statement on why infection control is particularly crucial for professional sports teams, such as the high level of contact and close proximity among players and staff.

[Our reply]

In response to your comment, we have changed the beginning of the Abstract. (page 2, lines 23-25)

• The line “The regular antigen testing scenario was more effective than the regular PCR testing scenario…” could be rephrased for clarity. For example: “Regular antigen testing was found to be more effective than PCR testing in reducing the number of infected individuals and associated costs.”

[Our reply]

As you suggested. Thank you. (page 2, lines 39-41)

• The costs associated with infection control are briefly mentioned, but providing a bit more specificity (e.g., what types of costs were included, or their relative importance) could strengthen the understanding of the analysis.

[Our reply]

As you suggested, we now give examples of costs, and we emphasize that some costs are in a trade-off relationship and it is important to find the optimum. (page1, line 25-30)

• In line 20, change “Infection control for players and team staff is an important issue” to “Infection control among players and team staff is a critical concern.”

[Our reply]

The sentence has been removed.

• In line 23, “operating costs” could be rephrased as “operational costs” for consistency with professional terminology.

[Our reply]

The sentence has been removed.

• Double-check the sentence structure and flow for grammatical clarity, particularly where figures and comparisons are being made.

[Our reply]

In response to this suggestion, we have redoubled our efforts by performing cross-checking among coauthors and by engaging a native-English-speaking professional science editor to read the manuscript.

Introduction

• In line 43, change “the 2019 corona virus” to “The 2019 coronavirus (COVID-19)” for accuracy and clarity.

[Our reply]

In response to the suggestion below, this sentence has been removed.

• In line 50, “quarantine asymptomatic infected individuals” could be clarified as “quarantine individuals with asymptomatic infections.”

[Our reply]

As you suggested. (page 2, line 54)

• In line 73, instead of “frequent low-sensitivity testing is effective,” consider rephrasing to “frequent testing using lower-sensitivity methods have been shown to be effective…”

[Our reply]

As you suggested. (page 3, lines 81-82)

Suggestions:

1. In line 43, consider specifying the timing of the outbreak (e.g., "In response to the global outbreak of the COVID-19 pandemic in early 2020") to provide a precise reference and remind the reader of the timeline.

[Our reply]

As you suggested. (page 2, line 47)

2. When listing current infection control measures (lines 47-49), you could expand briefly on why these measures alone may not be sufficient for professional sports settings, which helps justify the need for frequent testing protocols.

[Our reply]

We have added a sentence on page 3, lines 55-59:

“Professional sports teams, of course, earn their incomes by holding events. The spread of infection among teams results in various costs, including reduced attendance due to star players being absent because of infection, and loss of profits due to the cancellation of games because of large-scale infections. Infectious disease control is therefore considered to be a very important task.”

3. The phrase in line 55, "This testing protocol, however, was not adopted based on quantitative evaluation," is an important point but could be rephrased for clarity. You might say: "However, the current testing protocols have not been quantitatively evaluated to determine their effectiveness, raising questions about their actual impact on infection control."

[Our reply]

Thank you. As you suggested. (page 3, lines 64-66)

4. In lines 64-65, when introducing antigen testing as an alternative, you could emphasize the potential for this method to balance cost and frequency more explicitly: "To achieve a balance between cost savings and infection control, antigen testing with rapid results is being considered as a feasible alternative."

[Our reply]

As you suggested. (page 3, lines 72-74)

5. While the introduction touches on existing studies in lines 71-74, it would be beneficial to highlight the gap more explicitly, such as: "Despite these studies, there is a lack of research specifically addressing the dynamics and economic considerations of infection control within professional sports teams."

[Our reply]

As you suggested. (page 3, line 83-85)

6. In the last paragraph, the study objective could be stated more directly. For example: "This study aims to quantitatively assess the cost-effectiveness of different testing strategies for professional sports teams, focusing on the trade-offs between infection control, testing frequency, and associated costs."

[Our reply]

As you suggested. (page 3, lines 87-89)

Material and method

The "Materials and Methods" section is comprehensive, structured, and provides a clear methodology for the infectious disease dynamics model related to COVID-19. The use of a Monte Carlo simulation with 10,000 iterations and the application of an agent-based model add credibility to the analysis. The method for calculating the probabilities and the approach for discretizing the population dynamics shows thoroughness in modeling real-world scenarios.

[Our reply]

This is a summary of the section, and no revisions are required.

Suggestions:

1. While the details are comprehensive, the section could benefit from clearer subheadings or divisions to separate different aspects of the methodology, such as "Model Setup," "Mathematical Equations," "Simulation Details," and "Testing Scenarios." This would improve readability and help readers locate specific information more quickly.

[Our reply]

We have added a few subtitles. (page 4, line 107, page 5, line 137)

2. While the sensitivity values for the antigen test are based on PCR testing percentages, it would be helpful to include a brief explanation or rationale for these specific values to enhance transparency.

[Our reply]

We added a reference to show that the sensitivities in our simulations were close to those observed in J.League clubs (REF: 14). We have added a sentence to show this. (page 8, lines 232-233)

3. The reference to Table 2 is clear, but a brief summary of its contents would enhance understanding without the need to look at the table directly. Including more information in the text about the testing frequencies and reading times might help provide a quick reference for readers.

[Our reply]

We have added a brief summary of Table 2. (page 10, lines 255-260)

4. While some assumptions (like PCR's 0-day reading time) are mentioned, further elaboration on the implications of these assumptions or limitations (e.g., the generalizability of results beyond the J.League or other professional settings) would strengthen the section. Discussing the limitations of the data or the potential variations in real-world conditions would add depth.

[Our reply]

We agree that it is important to discuss the limitations of our study. Reviewer 1 also pointed out the importance of this. We consider that these limitations should be mentioned not in the Materials and Methods, but in the Discussion. We have added some limitations at the end of the Discussion (page 29, lines 800-821).

5. Although the text references figures (e.g., probability calculations), directly integrating these values or their implications within the paragraph could improve the overall flow and understanding. Including short summaries of results or anticipated findings from each scenario would make the section more engaging.

[Our reply]

We have added a brief summary at the end of each section. (page 18, lines 487-489, page, 20 lines 530-533, page 22, lines 580-582)

6. The section uses both “individuals” and “members” interchangeably, which could cause slight confusion. Consistent terminology would ensure clarity throughout.

[Our reply]

Except in the case of the first “member” (page 4, line 100), all “members” have been replaced by “individuals”.

7. While the section provides a detailed explanation of the formulas, it may be overwhelming for some readers unfamiliar with mathematical modeling. Adding short, summarized explanations or visual aids (e.g., flowcharts) could help make the calculations more accessible.

[Our reply]

We have added a rough outline of the simulation and cost calculations. Please see our new Figure 2.

8. The text assumes specific costs (e.g., quarantine cost per day, match postponement cost) but could benefit from a more explicit discussion of these assumptions and their implications. For example, acknowledging the variation in daily wages or stadium costs across different regions would provide context and increase the validity of the results.

[Our reply]

This is an important suggestion, but we do not think it is a suitable topic for the Materials and Methods. We now argue the limitations of our study at the end of the Discussion (page 29, lines 795-816). Please also see the Appendix A2 for the cost of the different players’ annual salaries; this calculation was conducted as one of the sensitivity analyses.

9. The decision to exclude the costs associated with death or infection treatment is justified, given the low risk for healthy individuals. However, mentioning the potential impact of long COVID or other complications that could affect professional athletes would provide a more comprehensive analysis of the possible outcomes.

[Our reply]

This is also an important point, but it was too complicated for us to consider in our simulations. We have now noted this important point, and we have also noted the fact that we did not consider it. (page 13, lines 359-363).

Discussion

While the study is thorough, there are several areas that could be strengthened:

• Although the model provides valuable insights, its applicability to real-world scenarios could be enhanced by incorporating additional variables, such as community transmission rates and individual behaviors outside of team activities.

[Our reply]

This point is important. We discuss it in the limitations section at the end of the Discussion (page 29, lines 800-821).

• The impact of testing protocols on player and staff mental health, particularly in high-pressure environments like professional sports, could also be addressed. This aspect is increasingly recognized as critical in managing public health crises.

[Our reply]

This is also an important point. We discuss it in the limitations section at the end of the Discussion (page 29, lines 800-821).

• Future research could explore the long-term implications of these testing strategies, particularly as new variants emerge and vaccination rates change.

[Our reply]

We refer to this in the limitations section of the Discussion. (page 29, lines 800-821)

We would like to thank you again for your many helpful comments.
